# Rethinking elite sport as high-stakes work: an organizational perspective on well-being and sustainable performance

**DOI:** 10.3389/fspor.2026.1924093

**Published:** 2026-08-21

**Authors:** Katrina Monton, Tanya Gulati, Eduardo Alonso Hernández Leiva

**Affiliations:** Department of Applied Psychology, Rutgers University, New Brunswick, NJ, United States

**Keywords:** elite sport, high-stakes work, organizational culture, organizational sport psychology, sustainable performance, well-being

## Abstract

Elite sport is characterized by chronic performance pressure, uncertainty, continual evaluation, and significant physical, psychological, relational, and occupational consequences. Although sport psychology has increasingly recognized the importance of organizational environments, athlete performance and well-being continue to be understood primarily through an individual lens. This perspective conceptualizes elite sport as a form of high-stakes work, shifting responsibility for sustainable performance and well-being beyond the individual athlete to the organizations that shape high-performance environments. We outline the defining characteristics of high-stakes work, identify the contextual conditions that distinguish elite sport, and examine how organizational infrastructure developed in comparable high-stakes domains may inform elite sport. We argue that communication systems, psychological safety, workload management, leadership, and organizational accountability should be understood as organizational conditions that support both athlete well-being and sustained performance. Conceptualizing elite sport as high-stakes work provides a systems-oriented framework for advancing research, practice, and organizational responsibility in elite sport.

## Introduction

Elite sport is frequently idealized as a domain of excellence and achievement, yet those working within these environments often contend with chronic evaluation, intense public visibility, uncertainty, relational interdependence, and high personal stakes (1). Careers may hinge on milliseconds, selection decisions, injury status, or fluctuations in performance that unfold under constant scrutiny (2, 3). Athletes frequently navigate unstable career trajectories, physical risk, and demanding interpersonal dynamics while being expected to perform with consistency and resilience (4, 5).

Over the past several decades, sport psychology has made substantial contributions to understanding performance and athlete well-being. Research on motivation, coping, resilience, mental toughness, attentional control, emotional regulation, and psychological preparation has significantly shaped both scholarship and practice within elite sport (4, 6, 7). While much of this work has focused on strengthening athletes' capacity to adapt and perform, scholars have increasingly turned their attention to the environments in which athletes operate, including coaching culture, psychological safety, athlete voice, organizational health, and thriving sport environments (2, 8–14). Yet dominant narratives continue to individualize distress, emphasizing resilience and mental toughness while obscuring the organizational conditions that shape athlete well-being and performance (14–16).

The central proposition of this perspective is that conceptualizing elite sport as high-stakes work shifts greater responsibility for sustainable performance and well-being to the organization, alongside the individual athlete. The contribution is not simply that organizational context matters, but that the high-stakes work lens reframes elite sport as an organizational system with responsibility for creating the conditions under which sustainable performance and well-being can be achieved.

This conceptual shift also opens the door to learning from other high-stakes domains. In industries such as aviation, healthcare, and nuclear power, sustained performance depends not only on individual capability, but also on the organizational systems, infrastructure, communication, leadership, and accountability that support it (17–19). Viewing elite sport through the same lens shifts the question from how athletes can better adapt to demanding environments to how organizations can deliberately create the conditions that enable sustainable performance and well-being.

While individual psychological preparation remains essential, other high-stakes domains have recognized that sustained performance depends on organizational environments that support communication, recovery, learning, and human functioning under pressure, conditions elite sport is only beginning to address (20–22). Although this perspective draws primarily on Western scholarship, athletes' experiences of harm and institutional protection vary considerably across national and cultural contexts (23). In settings where athletes face heightened structural vulnerability or fewer formal protections, organizational approaches that emphasize duty of care, psychological safety, and accountability may be particularly important.

Building on this premise, the paper examines organizational infrastructure developed in comparable high-stakes domains and considers how these principles might be adapted within elite sport. In aviation, medicine, and military settings, many of these practices emerged not simply as well-being initiatives, but as essential components of sustained performance under conditions of consequence (20–22, 24). We argue that conceptualizing elite sport as high-stakes work provides a framework for understanding these organizational practices as integral to sustainable performance and athlete well-being, rather than as ancillary supports. Recent evidence further suggests that healthier and more psychologically safe sport environments can support excellence and human flourishing (9, 11).

## Elite sport as high-stakes work

High-stakes work refers to occupational contexts characterized by sustained pressure, uncertainty, continual evaluation, and the potential for significant physical, psychological, relational, financial, or reputational consequences for individuals and organizations (25, 26). Conceptually, high-stakes work draws on scholarship examining high-risk occupations, extreme work contexts, and high reliability organizations, each of which highlights different dimensions of work performed under consequential conditions (22, 25–27).

The concept of high-stakes work overlaps with, but is distinct from, related constructs such as high-risk work and extreme work contexts. High-risk occupations primarily emphasize exposure to physical danger, whereas extreme work contexts encompass environments characterized by sustained physical, cognitive, and emotional demands under conditions of uncertainty and stress (25, 26). Scholarship on high reliability organizations has similarly focused on domains such as healthcare, aviation, military operations, and emergency response, where failures may carry catastrophic consequences (22, 27).

High-stakes work should also be distinguished from work characterized primarily by high-performance expectations. While many occupations require exceptional expertise or sustained excellence, high-stakes work is differentiated by the magnitude of the consequences associated with performance, the uncertainty under which work is performed, and the organizational conditions required to sustain functioning over time. All high-stakes work demands high performance, but not all high-performance work is inherently high stakes.

Together, these literatures capture important dimensions of work performed under consequential conditions but do not fully capture the broader constellation of chronic performance pressure, precarity, continual evaluation, public visibility, and heightened psychological, relational, and physical demands that characterize high-stakes work in elite sport.

In the present perspective, we conceptualize high-stakes work as occupational contexts characterized by chronic performance pressure, significant consequences, uncertainty, and sustained physical, psychological, and relational demands. These defining characteristics should be understood as dimensions rather than binary categories, such that occupations and organizational contexts may differ in the extent to which they exhibit each characteristic.

### What distinguishes elite sport as high-stakes work?

For the purposes of this perspective, elite sport refers to athletes competing at the international and world class level, broadly corresponding to Tier 4 and Tier 5 within McKay et al.'s (28) Participant Classification Framework, for whom sport represents a primary occupational commitment. We selected McKay et al.'s framework because it provides a standardized, objective, and sport inclusive approach to classifying athlete caliber based on training volume and competitive performance across diverse sporting contexts. Compared with earlier approaches, which were developed primarily within sport psychology and incorporate a wider range of indicators, including experience and competitiveness of the sporting domain (29), McKay et al.'s framework was specifically designed for consistent application across sport science and sports medicine research. Its utility has since been further supported through evidence demonstrating fair to substantial inter-rater reliability and moderate to almost perfect intra rater reliability when applied across published sport and exercise science studies (30).

For many athletes at this level, sport functions not simply as competition but as work: the primary occupation through which they earn a livelihood, organize their lives, and pursue future opportunities (3, 31). Athletic careers at this level are often short, intense, and all encompassing, demanding near total investment of physical, psychological, and relational resources (31). Consistent with McKay et al.'s (28) classification criteria, athletes at Tier 4 and Tier 5 undertake maximal or near maximal training volumes relative to their sport's norms. Combined with competition and travel obligations, these demands typically constitute full time occupational hours, regardless of whether financial compensation reliably reflects this level of investment.

At these elite levels, sport exhibits each of these defining characteristics and represents a distinctive configuration of high-stakes work. While chronic performance pressure, significant consequences, uncertainty, and sustained physical, psychological, and relational demands characterize many high-stakes occupations, elite sport intensifies these conditions through continual evaluation, public visibility, career precarity, early developmental entry, and profound identity investment. The sections that follow examine these defining, interrelated characteristics before considering the contextual conditions that distinguish elite sport from many other high-performance occupations.

#### Chronic performance pressure

Elite athletes perform under chronic performance pressure arising from continual training and competition, where exceptionally small differences in performance may influence competitive success and career progression (1, 2). Unlike occupations characterized by acute episodes of high pressure, elite sport requires sustained performance over extended periods, often across seasons and Olympic or World Championship cycles. Athletes are also expected to maintain performance despite injury, illness, selection uncertainty, and other setbacks that accompany elite competition (32, 33). These demands make pressure persistent rather than episodic. Chronic performance pressure therefore reflects not isolated moments of intense competition but the cumulative demands of sustaining elite performance over time.

#### Significant consequences

Athletes and sport organizations operate within systems where small errors may carry substantial physical, psychological, financial, and reputational consequences, while performance outcomes remain subject to continual evaluation by coaches, organizations, media, sponsors, and the public (1). The stakes extend well beyond competitive outcomes. For athletes at this level, sport often serves as both a livelihood and a primary source of identity, belonging, and future possibility, following years or decades of intensive investment beginning in childhood or adolescence (5, 31). Consequently, performance outcomes may influence not only competitive success but also occupational security, financial stability, identity, and future opportunities beyond sport. The consequences of participation may also extend well beyond athletic careers, including increased risk of long-term physical and mental health concerns, identity disruption during retirement and career transition, and enduring impacts on quality of life (33–35).

#### Uncertainty

Elite athletes must continually make decisions under conditions of uncertainty. Performance outcomes remain inherently unpredictable, while injuries, deselection, contract renewal, funding decisions, and career longevity are often outside an athlete's direct control (36, 37). Uncertainty also extends to the long-term health consequences of participation, as many medical decisions, including return-to-play following following injury, like concussion, must be made despite incomplete knowledge regarding future long-term outcomes (38–40). These multiple forms of uncertainty reinforce the high-stakes nature of elite sport.

#### Sustained physical, psychological, and relational demands

Participation in elite sport requires sustained physical, psychological, and relational investment over many years. Athletes must continually balance intensive training loads, injury management, travel, recovery, and competition alongside ongoing psychological demands, including performance anxiety, perfectionism, and public scrutiny (33). These demands are experienced within complex relational systems involving coaches, teammates, practitioners, administrators, and governing bodies, where power differentials, dependence, and interpersonal dynamics shape both performance and well-being (32, 41, 42).

### Contextual conditions that distinguish elite sport as high-stakes work

The defining characteristics described above are common across many high-stakes occupations. Elite sport is distinctive because several contextual conditions amplify how these characteristics are experienced. These conditions do not define high-stakes work itself; rather, they shape its intensity, persistence, and organizational implications within elite sport. We highlight five particularly salient contextual conditions: continual evaluation, public visibility, career precarity, early developmental entry, and profound identity investment.

#### Continual evaluation

Elite athletes operate within systems where performance outcomes remain subject to continual evaluation by coaches, organizations, selectors, sponsors, media, and the public (1). Unlike many occupations in which performance is reviewed periodically, evaluation in elite sport is continuous and directly influences selection, funding, competitive opportunities, and career progression. Increasingly, athletes are evaluated not only through formal organizational processes but also through ongoing public commentary across traditional and social media, where performances and decisions may be continuously observed, interpreted, and debated (43, 44). As a result, athletes experience persistent scrutiny that reinforces chronic performance pressure and heightens the consequences associated with success and failure.

#### Public visibility

Elite sport is also distinguished by its high degree of public visibility. Athletic performances frequently occur before spectators and media audiences and are increasingly documented, shared, and recirculated through digital and social media platforms, extending visibility well beyond the moment of competition (1, 43, 44). This visibility is not merely incidental to elite sport but constitutes a recognized occupational stressor associated with increased vulnerability to anxiety and depression (45). The persistence of digital media also limits athletes' ability to disengage from public scrutiny, as performances, decisions, and personal challenges may remain visible and open to ongoing commentary long after competition has ended, amplifying the reputational consequences of performance (46, 47).

#### Career precarity

The finite and precarious nature of elite athletic careers creates additional pressure. Performance, injury, deselection, and organizational decisions may substantially alter career trajectories (36, 37), leading athletes to perceive raising concerns or resisting harmful demands as carrying unacceptable personal and financial costs (32). Opportunities for selection, funding, contracts, and continued participation are often contingent on both performance and organizational decisions, leaving athletes with limited control over many aspects of their careers (3, 31). Consequently, athletes may be less likely to seek help, disclose injuries, or speak up about unsafe or unhealthy environments when doing so is perceived to jeopardize future opportunities (32, 48). Career precarity therefore reinforces athletes' dependence on organizational decisions, relationships, and systems of selection and support.

#### Early developmental entry

Unlike many high-stakes occupations that individuals enter as adults, elite sport frequently begins during childhood or adolescence, particularly in sports characterized by early specialization (e.g., gymnastics). Young athletes, many of whom enter high-performance systems during formative developmental periods, operate within relationships marked by pronounced power differentials, where coaches exercise considerable authority over training, selection, and career progression (32, 49). Early recruitment, intensive training demands, and prolonged immersion in high-performance sport may narrow opportunities for broader identity development, increasing the likelihood of identity foreclosure, a premature commitment to the athletic role that can make adaptation beyond sport more difficult (41).

#### Identity investment

Elite sport is further distinguished by the profound identity investment it often requires. Following years or decades of intensive participation beginning in childhood or adolescence, sport frequently becomes a primary source of identity, belonging, and future possibility (5, 31). For some athletes, this intensive investment may make athletic identity increasingly difficult to separate from the self, rendering transitions beyond sport particularly challenging following involuntary or premature career termination (3, 31, 34).

#### Reinforcing cultural narratives

The contextual conditions described above are further reinforced by cultural narratives that celebrate passion, sacrifice, resilience, and extraordinary commitment as defining features of elite sport. These narratives can obscure the occupational reality of elite sport, framing participation primarily as passion or privilege rather than as work performed under demanding organizational conditions. In doing so, they may normalize suffering and a win-at-all-costs mentality while shifting attention away from the organizational conditions and leadership practices that shape athlete experiences (15, 16). Research suggests that portraying workers as exceptionally passionate, self-sacrificing, or heroic increases assumptions that they willingly accept exploitation and reduces concern about unfair or harmful working conditions (50, 51). Applied to elite sport, these narratives may inadvertently legitimize excessive demands, discourage help-seeking, and reinforce organizational environments in which silence, overwork, and self-sacrifice become expected rather than questioned (41).

Together, these defining characteristics and contextual conditions provide a framework for conceptualizing elite sport as a distinctive configuration of high-stakes work. Table 1 summarizes the framework and its illustrative organizational implications.

**Table 1:** Defining characteristics and contextual conditions of elite sport as high-stakes work.

Dimension	Definition	Example in elite sport	Organizational considerations
Defining characteristics			
Chronic performance pressure	Sustained, cumulative demands of performing at a high level over extended periods, rather than isolated episodes of acute pressure.	An athlete maintaining Olympic-qualifying performance across a four-year Olympic cycle despite injury, illness, or selection uncertainty.	Workload monitoring, recovery planning, and organizational systems that support sustainable performance over time.
Significant consequences	Small errors or outcomes carrying substantial physical, psychological, financial, and reputational consequences.	A missed selection cutoff or fractional timing margin determining Olympic team membership and future funding.	Transparent decision-making, duty of care, and organizational accountability for high-stakes decisions.
Uncertainty	Decisions made under incomplete knowledge of outcomes, including long-term health consequences.	Return-to-play decisions following concussion made without full understanding of cumulative long-term risk.	Communication systems, shared decision-making, and psychologically safe environments for navigating uncertainty.
Sustained physical, psychological, and relational demands	Ongoing investment across training, recovery, competition, and complex relational systems over many years.	An athlete balancing training load, international travel, and coach–team dynamics across a decade-long career.	Coordinated interdisciplinary support, relational stability, and organizational resources that sustain long-term well-being and performance.
Contextual conditions			
Continual evaluation	Performance subject to ongoing, rather than periodic, assessment by multiple stakeholders.	Coaches, selectors, sponsors, and media evaluating performance after every competition rather than through annual review cycles.	Psychologically safe feedback processes, transparent evaluation systems, and supportive leadership.
Public visibility	Performance occurring before audiences and media, with successes and failures publicly documented and often permanently accessible.	A missed shot or visible performance error captured on broadcast television and circulated through social media.	Media support, communication strategies, and organizational approaches to managing public scrutiny.
Career precarity	Finite, injury- or selection-dependent careers that may discourage reporting concerns or seeking support.	An athlete choosing not to report an injury for fear of losing a roster position before Olympic Trials.	Independent reporting mechanisms, appropriate organizational protections, and cultures that reduce fear of speaking up.
Early developmental entry	Entry into high-performance systems during childhood or adolescence under pronounced power differentials.	A gymnast entering elite training pathways at six to eight years of age, years before reaching legal adulthood.	Safeguarding, independent oversight, and developmentally appropriate organizational support.
Identity investment	Occupational identity becoming closely intertwined with self-concept, particularly following involuntary exit from sport.	An athlete experiencing profound identity disruption following deselection or career-ending injury.	Career transition planning, identity development, and holistic support beyond competitive performance.

Conceptualizing elite sport as high-stakes work also requires clarifying the scope of the present perspective. Although many occupations, including performing arts, ballet, surgery, investment banking, military service, and emergency response, share characteristics associated with high-stakes work, the present perspective is concerned specifically with elite sport. The purpose of the present perspective is not to determine which occupations qualify as high-stakes work, but rather to examine what becomes visible when elite sport is understood through this lens. Although the defining characteristics of high-stakes work may be present to varying degrees across different occupations, the present paper argues that elite sport represents a distinctive configuration of these defining characteristics together with the contextual conditions described above (1, 2, 5, 31).

Conceptualizing elite sport in this way does not suggest that it is uniquely high stakes or directly equivalent to aviation, medicine, military operations, or other occupations where errors may immediately threaten public safety or result in catastrophic loss of life. Instead, it highlights similarities in the organizational conditions under which sustained performance must be achieved while recognizing important differences in the nature, governance, and consequences of those conditions (20–22, 27). These differences extend beyond governance. Unlike aviation, healthcare, and many military contexts, elite sport is fundamentally organized around competition rather than service delivery, participation is formally voluntary despite substantial structural and developmental pressures, and organizations operate within competitive systems in which performance outcomes are inherently relative rather than standardized. As a result, organizational practices cannot be transferred wholesale across contexts but instead require adaptation to the distinctive realities of elite sport.

A further distinguishing feature concerns the position of the athlete within the organizational structure itself. In aviation, medicine, and other high reliability domains, multidisciplinary teams collaborate to deliver a service or protect a third party, such as passengers, patients, or the public, from harm. In elite sport, by contrast, the athlete is simultaneously the focal point of performance and the primary recipient of support provided by the multidisciplinary team surrounding them (1). Practitioners are therefore required to collaborate not only with one another but also in service of the athlete, a relationship that may shape authority, accountability, communication, and decision making in ways that further distinguish elite sport from other high stakes domains. This relationship may also create distinctive tensions because practitioners such as team physicians and sport psychologists often hold dual responsibilities to both the athlete and the sport organization, requiring them to balance athlete welfare with organizational performance interests (52). This distinctive practitioner-athlete relationship introduces organizational dynamics that are uncommon in many other high reliability settings and provides an additional reason why organizational practices and infrastructure developed in other high reliability domains cannot be transferred directly into elite sport without careful adaptation.

Elite sport also differs from many comparable high-stakes domains in its governance structures. Medicine, aviation, and many military systems operate under independent licensing bodies, mandatory reporting systems, accreditation processes, and established mechanisms of organizational accountability. By contrast, elite sport remains comparatively fragmented and largely self-regulating, with governing bodies often simultaneously responsible for athlete performance and athlete welfare (53, 54).

The value of conceptualizing elite sport as high-stakes work therefore lies not in suggesting that elite sport is uniquely demanding, but in shifting the unit of analysis from athlete adaptation toward the organizational conditions required to support sustainable performance under chronic pressure, uncertainty, and significant consequence. This perspective invites questions not only about how athletes adapt to demanding environments, but also about how those environments are designed, led, and sustained. Although athletes remain the primary focus of the present perspective, these organizational conditions extend beyond athletes alone. Elite sport is enacted within interconnected systems that include coaches, performance support staff, leaders, administrators, officials, parents, governing bodies, and the broader communities surrounding high-performance sport, each of whom both shapes and is shaped by the organizational environment. Conceptualizing elite sport through a high-stakes work lens therefore has implications that extend across the wider elite sport system.

### Implications for the broader elite sport system

The preceding sections have focused on athletes as the primary performers within elite sport, arguing that sustainable performance and well-being depend on organizational infrastructure rather than individual adaptation alone. Yet if this infrastructure is genuinely organizational in nature, responsibility cannot rest with athletes alone. Elite sport is enacted through interconnected relationships among athletes, coaches, performance support staff, leaders, officials, parents, governing bodies, and the broader communities that sustain high-performance sport. The organizational conditions that shape athlete well-being and performance are created, maintained, and experienced across this wider system (13, 55). A high-stakes work framework therefore implies a shift from athlete-centered to human-centered organizational design, in which the infrastructure required to support sustainable performance is understood as a shared organizational responsibility rather than a resource allocated to athletes alone.

Coaches occupy their own high-stakes position within this system, subject to continual evaluation, job insecurity, and organizational demands that parallel those experienced by athletes, yet with comparatively little organizational or empirical attention paid to their well-being (56, 57). Officials and referees face similarly under-recognized demands, including direct exposure to verbal and physical abuse that carries measurable mental health consequences (58). Parents, particularly of young athletes entering elite sport, experience their own organizational, financial, and relational pressures while supporting their children's participation (59). These pressures illustrate that the consequences of organizational conditions extend beyond athletes to the broader high-performance sport system, even though these groups typically fall outside existing duty-of-care frameworks.

Other high-stakes domains offer a precedent for this broader view of organizational responsibility. Military organizations, for example, have developed extensive family readiness systems grounded in the recognition that service member performance and retention are inseparable from the well-being of the family system that supports them (60). This infrastructure is not treated as ancillary support extended out of goodwill, but as a performance-relevant investment in the conditions that allow individuals to function sustainably within demanding systems. Elite sport organizations have rarely extended comparable thinking beyond the athlete, despite operating within similarly interdependent systems. The point is not that elite sport should replicate military family support programs, but that organizational responsibility can extend beyond the individual performer to the wider system that enables sustained performance. Recognizing coaches, officials, parents, and communities as embedded within, rather than peripheral to, the high-stakes work of elite sport suggests that organizational infrastructure designed only around athletes will remain structurally incomplete.

### Developmental and equity considerations

Importantly, this perspective does not assume that athletes experience high-stakes work in the same way. Developmental stage, gender, race, disability, migration status, socioeconomic position, and the resources available within particular sport systems shape athletes' exposure to risk, access to protection, and opportunities to exercise voice within organizations (41, 53, 61).

Developmental considerations are particularly important because entering elite sport during childhood or adolescence may limit athletes' capacity to evaluate organizational conditions, recognize inappropriate practices, or safely challenge harmful environments. Dependence on coaches, organizations, and selection systems may further constrain athletes’ willingness or ability to exercise voice, increasing reliance on organizational safeguards (32, 49). These developmental realities distinguish elite sport from many occupations entered after adulthood and further reinforce the importance of organizational responsibility.

Structural inequities may further intensify the experience of high-stakes work. Athletes from historically marginalized groups, including women, para-athletes, racialized athletes, migrant athletes, and those competing within under-resourced sport systems, may encounter additional barriers to inclusion, protection, reporting, and access to organizational support (23, 53, 61). For example, research in para sport suggests that disability-related stigma, accessibility challenges, classification systems, and greater dependence on coaches, caregivers, and support personnel may create additional power asymmetries and organizational vulnerabilities that shape athletes' experiences (62, 63). These examples illustrate that although the defining characteristics of high-stakes work may be shared across elite sport, the organizational conditions required to support sustainable performance are not experienced uniformly. Accordingly, organizational responsibility should extend beyond universal principles of duty of care and psychological safety to include attention to the structural and contextual realities under which athletes participate.

Although this perspective draws primarily on Western scholarship, organizational structures, governance arrangements, and athletes' experiences of harm vary considerably across national, cultural, and institutional contexts (64). This imbalance is reflected in the safeguarding literature itself. Research on interpersonal violence in sport has focused predominantly on Global North countries, with the first prevalence study among high-performance athletes in Brazil appearing only recently (23). These differences also shape how risks are managed. China's centralized elite sport system, for example, concentrates training, resource allocation, and career decisions within state sport organizations, creating patterns of organizational oversight that differ substantially from the more decentralized pathways common in North America and Europe (65). Japan's prefecture-based talent identification and development system reflects a different organizational model, embedding athlete development within regional sport councils and school systems rather than predominantly club- or collegiate-based pathways (66). Together, these examples illustrate that although the defining characteristics of high-stakes work may be shared across elite sport systems, the organizational structures through which they are experienced, and the mechanisms required to support athlete well-being and accountability, vary across national contexts. Accordingly, organizational approaches emphasizing duty of care, psychological safety, and accountability should be understood as contextually adaptable principles rather than uniform solutions.

## Beyond individual adaptation: organizational responses to high-stakes work

Understanding what equivalent infrastructure might look like within elite sport is aided by examining how comparable high-stakes domains have approached the same challenge. Across aviation, medicine, and military settings, recognition that performance failures often emerge from organizational rather than purely individual factors has led to the development of communication systems, reporting mechanisms, workload protections, leadership frameworks, and psychological support structures designed to support both safety and performance (20, 24, 27, 67). In aviation, this has included Crew Resource Management and incident reporting systems (22, 27). Medicine has emphasized structured debriefing, workload management, and duty-of-care practices (20). Military organizations have developed formalized leadership and psychological support programs designed to sustain functioning under pressure (24, 67).

**Figure 1:**
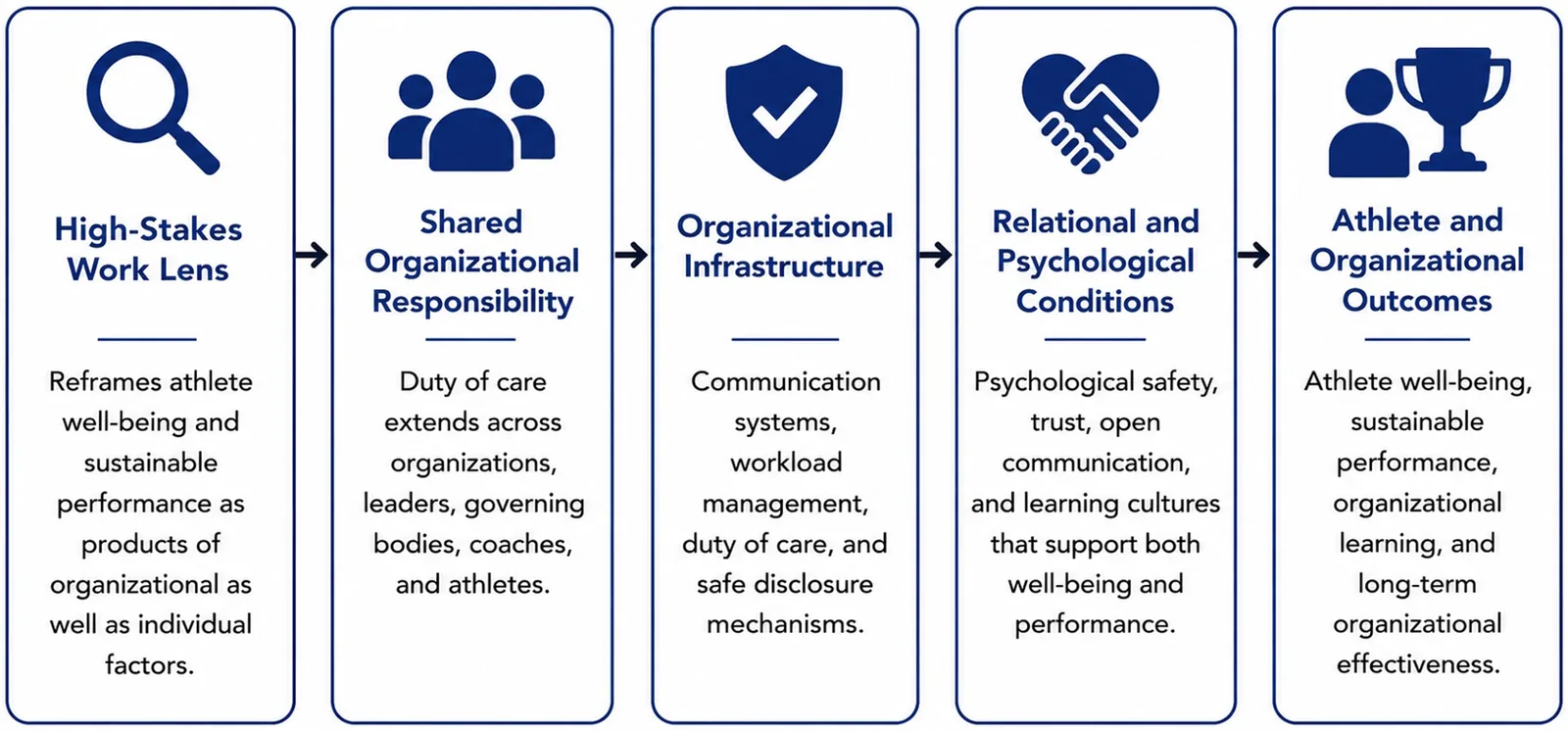
Reconceptualizing elite sport as high-stakes work. The figure illustrates how reframing elite sport as high-stakes work broadens attention beyond individual athlete adaptation to the organizational conditions and infrastructure that support sustainable performance and athlete well-being. The visual was created using a generative AI tool based on author-developed concepts and was reviewed and edited by the authors for accuracy and clarity.

Elite sport, by contrast, has been slower to develop comparable organizational infrastructure. Athlete mental health support, where it exists, is typically positioned as individual clinical provision rather than organizational conditions (10, 14, 68). Workload regulation, communication protocols, psychological safety audits, and duty of care frameworks remain inconsistent across sports and governing bodies (10, 69, 70). Mechanisms through which athletes can safely disclose concerns about training, interpersonal dynamics, or organizational practices without fear of selection consequences remain rare (68, 71, 72). The dominant model remains one in which athletes are expected to adapt to demanding systems rather than systems being designed to sustain the humans within them (14, 53). This matters not only for athlete well-being but for performance itself. Chronic psychological threat, fear-based climates, and the suppression of distress consume cognitive and emotional resources that would otherwise be available for learning, adaptation, and competitive execution (73, 74). Performance and well-being are often treated as competing priorities, yet both may depend on the same underlying organizational conditions (9, 11). Figure 1 illustrates how reframing elite sport as high-stakes work broadens attention beyond individual athlete adaptation to the organizational conditions and infrastructure that support sustainable performance and athlete well-being.

## Cultural norms and organizational learning

Elite sport cultures are often organized around ideals of sacrifice, discipline, and unwavering commitment to performance excellence. Athletes are socialized into environments that valorize pain tolerance, emotional control, and the prioritization of sport above competing personal needs or identities (53, 74, 75). Understanding why elite sport has been slow to adopt systems-level thinking requires examining these norms not simply as cultural values, but as organizational mechanisms that shape what is recognized, rewarded, and questioned within high-performance environments. Applications of the Masculinity Contest Culture (MCC) model suggest that high-performance sport normalizes suppressing distress, tolerating unhealthy workloads, and showing no weakness in order to maintain status and competitive opportunity (74, 76).

Practices that other high-stakes domains came to view as barriers to learning and adaptation, including silence around error, suppression of distress, and deference to authority, have often been interpreted within elite sport as markers of commitment, toughness, and competitive legitimacy (53, 74, 75). In aviation, silence around near-misses came to be understood as undermining safety and organizational learning, prompting the development of incident reporting systems (22, 27). In elite sport, comparable silence has more often been interpreted as resilience rather than a signal warranting organizational review (53, 74). Consequently, environments characterized by fear, silence, and the suppression of distress constrain organizational learning and ultimately undermine both human well-being and sustained performance (22, 77).

## Organizational conditions and sustained performance

Performance in elite sport is not solely an individual phenomenon. Research consistently demonstrates that athletes' experiences are shaped by coaching relationships, communication climates, role ambiguity, team culture, selection processes, and organizational stability (13, 55, 70). Psychological safety, the shared perception that individuals can express concerns, admit mistakes, and communicate openly without fear of humiliation or exclusion, is a foundational condition for learning, adaptability, and collective functioning across high-stakes environments (77). Within elite sport, psychologically safe coaching climates, relationally supportive team environments, and organizations characterized by clear communication and structural stability have been associated not only with athlete well-being but also with adaptive functioning, learning orientation, and sustained performance under pressure (55, 70, 78, 79). Conversely, fear-based leadership, chronic instability, and relational insecurity may undermine communication, increase anxiety, and constrain both learning and performance (70, 79). Together, these findings reinforce a central proposition of the present perspective: the organizational conditions that support athlete well-being are also the conditions that enable sustained performance.

## Implications for practice and organizational design

When psychological preparation is framed primarily as individual adaptation to demanding environments, organizational contributors to distress may remain insufficiently examined. Athletes may be taught to cope more effectively with chronic instability, excessive workload, relational insecurity, or psychologically unsafe environments without equivalent attention being paid to the systems producing these conditions. Similar shifts have occurred in other high-stakes domains, where performance and well-being came to be understood as products of both individual capability and organizational conditions. Accordingly, psychological preparation should extend beyond individual mental skills to consider how organizational conditions, leadership dynamics, cultural norms, and relational environments shape athlete functioning. This perspective does not require abandoning traditional mental skills approaches; rather, it involves integrating them within a broader understanding of how high-performance systems function. Psychological resilience remains essential, but it is likely to be more sustainable when supported by trust, psychological safety, ethical leadership, and organizational cultures that value both performance and humanity.

Conceptualizing elite sport as high-stakes work also carries direct implications for sport organizations, governing bodies, and leadership structures responsible for shaping performance environments. If athlete well-being and sustainable performance depend in part on organizational conditions, responsibility for supporting those outcomes necessarily extends beyond the athlete alone. From this perspective, organizational infrastructure becomes a performance-critical feature of elite sport rather than a secondary concern. Unlike medicine or aviation, elite sport lacks a single regulatory authority capable of implementing system-wide reforms. Instead, its fragmented governance across clubs, national governing bodies, international federations, and professional organizations means meaningful organizational change will require coordinated action across multiple levels of the sport system.

At a practical level, governing bodies and sport organizations may need to embed duty of care within organizational policies and performance systems (10), not only responding to harm after it occurs but proactively examining how workload expectations, communication structures, selection systems, and leadership norms shape both well-being and performance. Sport may similarly benefit from developing organizational infrastructure that other high-stakes domains have found essential, including communication protocols, workload regulation, safe disclosure mechanisms, and leadership accountability systems. Implementing these changes will not be straightforward. Governance remains fragmented across clubs, federations, and national bodies, and the organizations responsible for performance are often the same ones responsible for athlete welfare, creating potential conflicts of interest that may undermine independent oversight (53, 54). Athletes considering disclosure may face genuine risks of deselection (71, 72), and many sport systems lack the resources needed to build the organizational infrastructure that meaningful reform requires.

## Conclusion

Individual-focused frameworks in sport psychology remain essential but incomplete. They cannot fully account for the organizational conditions within which athletes perform or for the structural realities of elite sport as a form of high-stakes work. Other high-stakes domains have demonstrated that sustained performance depends not only on individual capability but also on the organizational systems that support communication, learning, accountability, and human functioning under pressure.

Conceptualizing elite sport as high-stakes work extends this perspective to elite sport by shifting the unit of analysis from the athlete alone to the organizational conditions under which sustainable performance is created, supported, and sustained. Under this lens, athlete well-being and sustained excellence are not competing priorities but outcomes that depend on the same organizational foundations.

## Data Availability

The original contributions presented in the study are included in the article/Supplementary Material, further inquiries can be directed to the corresponding author/s.
